# Nutritional Evaluation of Buns Developed from Chickpea-Mung Bean Composite Flour and Sugar Beet Powder

**DOI:** 10.1155/2022/6009998

**Published:** 2022-03-18

**Authors:** Bayan Zh. Muldabekova, Gulzhanat A. Umirzakova, Zhazira R. Assangaliyeva, Pernekul M. Maliktayeva, Ainur A. Zheldybayeva, Madina A. Yakiyayeva

**Affiliations:** ^1^Department of Technology of Bread Products and Processing Industries, Almaty Technological University, Almaty, Kazakhstan; ^2^Higher School of Technology of Food and Processing Industries, West Kazakhstan Agrarian and Technical University Named after Zhangir Khan, Uralsk, Kazakhstan; ^3^Department of Standardization and Veterinary Sanitation, International Taraz Innovation Institute, Taraz, Kazakhstan; ^4^Department of Food Safety and Quality, Almaty Technological University, Almaty, Kazakhstan; ^5^Research Institute of Food Technologies, Almaty Technological University, Almaty, Kazakhstan

## Abstract

The research was aimed at developing recipes for buns studying the nutritional value of securities. In the work, an assortment of bakery products was developed from flour, composite mixtures of leguminous crops and dry powders of sugar beets. As a result, bakery products with useful properties and improved qualities were obtained. In the recipe, sugar was completely replaced by dry powders of sugar beet. The optimal combination for making a bun from composite flour and dry sugar beet powder was 10% chickpea and 5% mung bean flour with 9.23 g of dry sugar beet powder added per 100 g flour. Physical and chemical indicators, including mineral elements, vitamin composition, and safety indicators, were determined. It was proven that the use of composite flour from leguminous crops contributes to a contraction of the technological process of the production of bakery products, reducing the time needed for dough preparation and baking. The use of technology for obtaining bakery products and recipes in production allows expanding the range of bakery products, reducing the duration of the technological process of production, improving the quality of finished products, and increasing labour productivity. It also helps to improve the socioeconomic indicators of bakery and confectionery enterprises.

## 1. Introduction

Recently, the entire population of Kazakhstan has taken an interest in products for a healthy lifestyle. The bakery industry today is one of the most rapidly developing sectors in Kazakhstan [1]. At the present stage of the development of Kazakhstan, ensuring the stable operation of enterprises for the production of competitive products is a task of paramount importance for managers of all levels. The most important characteristic of management at all levels is the organization and efficiency of production [2].

In a market economy, the only enterprise that survives is the one that most competently and completely determines the requirements of the market, creates and organizes the production of products that are in demand, and provides high income for highly qualified workers [3–5]. Reducing production costs, rational use of material resources, achieving higher economic indicators, and, above all, increasing labour productivity and production efficiency, which in turn reduces costs, are the most important and urgent tasks of production management workers. To solve the problem, it is of great importance to improve management to increase its efficiency and master the methods of effective production management, as well as to calculate and compare indicators of the enterprise's production efficiency [6–8].

Bread and bakery products have always been and remain one of the main food product categories consumed by the population. Bakery products are one of the staple foods in the human diet [9–12].

Bakery products are the most important human food. Daily bread consumption in different parts of the country ranges from 150 to 500 per capita. Bread contains many essential nutrients; these include proteins, fats, carbohydrates, vitamins, minerals, and dietary fibre [13–15].

In addition to traditional methods of dough preparation (using fermented, unleavened, thick and liquid yeasts), bakeries also use more advanced methods (reducing the duration of fermentation and using enhancers and additives).

The main direction in improving the quality of bakery production and increasing the efficiency of enterprises is the introduction of innovative dough technologies, expanding the range of dietary, medicinal, and children's products. For the preparation of these products are flour mixtures with bran, unrefined and crushed grains, sunflower, sesame seeds, vitamin-mineral components, biologically active additives, etc., applied [16–18].

An acute disease is a deficiency of vitamins, in particular, obesity C (in 60-70% of the population of Kazakhstan), folic acid (70-80%) and minerals: iron (20-40%), cholesterol (40-60%), and iodine (up to 70%). Such deficiencies reduce the functional activity of the immune system and constitute risk factors for a large number of common chronic diseases [19, 20].

Grain crops are grown in agriculture. Their processed products or fruits themselves are used as primary and secondary raw materials in the bakery industry. The main sources of raw materials are flour, water, salt and yeast. These are the most important components in the recipe of bakery products. Additional sources of raw materials are used to increase the nutritional value and improve the taste and aroma of bakery products. These include sugar and sugar-containing foods, fats, milk and dairy products, eggs and egg products, malt, fruit plants, various nuts, spices, and additives [21, 22].

Wheat, buckwheat, triticale, oats, barley, and corn cereals used to make flour. Legumes (soy and peas) are used as additives that increase the nutritional value of bakery products. Essential oil crops (cumin, fennel, sunflower, etc.) add a special taste and aroma to bakery products. One of the most important cereals is wheat. Wheat flour proteins turn into tissue when mixed with water. It is often used in the manufacture of bakery and pasta products and cereals. Products made from whole grains and whole grains are usually used for dietary nutrition [23–27].

The results of literary studies show that effective technology is needed to ensure a product with high quality and useful properties. It is also necessary to develop technologies for producing sugar beet powders for use as sugar substitutes in the manufacture of bakery and flour confectionery products.

In this work, a range of bakery products was developed from composite flour mixtures of legumes and dried sugar beets. Their use will help to improve the quality and beneficial properties of sugar products, shorten the production process, ensure waste-free technology, increase labour productivity, and increase the socioeconomic indicators of bakery and flour confectionery enterprises.

## 2. Materials and Methods

### 2.1. Used Standard Methods and Raw Materials for Research

The methodological basis of the study was a systematic analysis of the technology used in the production of bakery products enriched with useful herbal ingredients. The following main tasks were performed sequentially: (1) selection and justification of the method for introducing herbal ingredients into the recipe for bakery products and (2) improvement of the technology used in bakery products by incorporating useful plant ingredients. The theoretical basis of the research consisted of general scientific and special research methods, methods of system analysis, and experimental planning. The objects of research were wheat flour of the first grade, chickpea and mung bean flour, and sugar beet.

In the work, the following indicators of the raw materials used and the resulting assortments of bakery products were investigated: organoleptic indicators by GOST 5667-85, mass fraction of moisture by GOST 21094-75, mass fraction of fat by GOST 5668-68, mass fraction the proportion of protein by GOST 10846-91, and others.

The crude fiber content was also determined by the Wende method on a Fiwe-6 device [28]. Determination of the content of mineral elements consists of dry mineralization of the sample at 450°C, ash dissolution, and titration of the ash solution with Trilon B solution in the presence of an acidic chrome dark blue indicator. Ash content was then determined by the atomic absorption method. The content of heavy metals cadmium and lead was determined by the method of atomic absorption spectroscopy (АСС) on a spectrometer with electric atomization “KVANT-Z.ETA-T” with software.

Vitamins B1, B2, and PP were determined by capillary electrophoresis on a Kapel-105M “Lumex” device. The content of pesticides, including heptachlor, *α*-, *β*-, and *γ*-isomers of hexachlorocyclohexane (HCH), dichlorodiphenyl trichloromethylmethane (DDT), and its metabolites, was determined by gas-liquid chromatography. Additional methods and techniques were also used.

We used leguminous crops such as chickpeas and mung beans. Composite flour enriched with protein, dietary fibre, B vitamins, and macro- and microelements was obtained from them.

**Figure 1:**
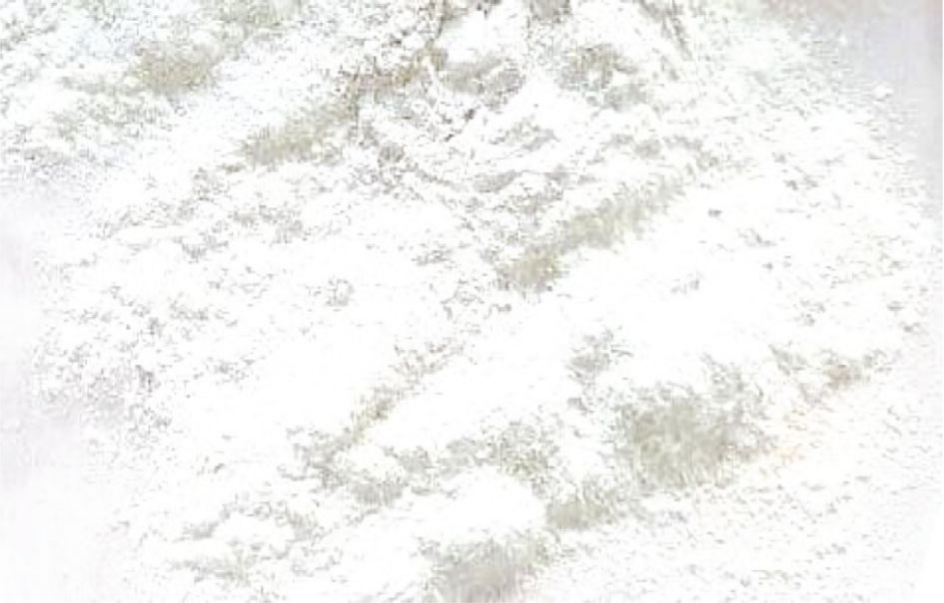
Dry sugar beet powder.

A method of drying sugar beet was developed, and a finely ground powder was obtained. Fresh sugar beet tubers were sliced thinly and dried without disturbing the structure on a Hurakan HKN-DHD10 dehydrator at a temperature of 70°C for 4.5–5 hours and subsequently crushed in an LZM-1M laboratory mill with a given stable particle size. The resulting sugar beet powder is shown in Figure 1.

The main raw materials for baking bread are first-grade flour, chickpea flour, mung flour, water, milk, sugar, sugar beet powder, shiver, butter, and an egg. Various assortments of rolls were received. Comparing the quality of the dough and cooking and baking times, the following assortments of buns were selected for further research (for one portion of laboratory baked goods):

The dough mass for one piece of bun was 50 g. In one portion (about 500 g) of laboratory baked goods prepared according to this recipe, an average of 10–12 buns were obtained.

Butter buns are buns made from sweet yeast dough. Buns can be prepared for every taste: plain or with raisins, poppy seeds, striser crumbs, sugar topping, etc. In this work, ordinary buns without filling were prepared. To eliminate sugar from the bun recipe, sugar beet powder was added. Dried sugar powder contains about 70% sugar and also 7.1% moisture. Sugar contains almost no moisture and leads to a thinning of the dough. The reason for this is that increasing the osmotic pressure in the liquid phase of the dough reduces the swelling of the flour colloids. Due to the high content of free water, the dough liquefies, although the total moisture content decreases. Therefore, compared with the sugar bun recipe, there was a difference in the amount of first-grade flour in the sugar beet powder bun recipe. Also, flour from legumes, especially chickpea flour, strongly absorbs moisture. For them, first-grade flour was added depending on the consistency of the dough. The recipes for the various bun formulations are shown in Tables 1 and 2.

To prepare the dough, Pakmaya dry yeast, which consists of the natural yeast *Saccharomyces cerevisiae* and the food emulsifier E-491, was used. Since *S. cerevisiae* is very active and of high quality, they reduce the rising time. The yeast is placed directly in warm milk for about 10–15 min for activation before incorporation into the dough and subsequent kneading. In the work, the dough was prepared using a dry Pakmaya yeast liquid sponge with a moisture content of more than 65% for 15 min. With the sponge method of preparing the dough, the products undoubtedly have a better taste and aroma, as well as more developed and better porosity than with the unpaired one. The sponge dough is highly hydrophilic, and it contains more colloids peptized by water; its viscosity and yield point, even immediately after kneading, are less than that of a bezoparny dough. This is because when kneading the sponge dough, the starch and proteins of the part of the flour that was in the dough have already undergone certain enzymatic and colloidal changes during the fermentation of the dough and are added to the dough in a finished state.

The buns were prepared in the following order (for one portion of baking):

The milk was warmed up to room temperature, and sugar and yeast were added to it. Then, about 25 g of flour was added, and all the ingredients were mixed. After that, the container was closed with a lid and placed in a proofing cabinet with a temperature of 30–32°C for 15 min. Then, the rest of the ingredients were added, and the dough was kneaded. Flour for samples no. 4–no. 10 was added depending on the mass of chickpea and muffin flour, as well as dry sugar beet powder. Then, the dough was sent to a proofing cabinet with a temperature of 35–36°C (proofing no. 1). After the dough rose to the desired volume, it was divided; that is, small balls of 50 g were molded from it and placed on a baking sheet. Then, it was sent to a proofing cabinet with a temperature of 35–36°C (proofing no. 2). When the balls were enlarged to the desired size, they were greased (using a cooking brush) with beaten egg yolk and placed in a Unox XFT133 convection oven preheated to 150–160 degrees. It is better to place the baking sheet in the middle of the oven, so that there is approximately the same distance to the top and bottom of the oven, to ensure even baking. The buns were baked at 180°C. The baking time depends on the composition and properties of the raw materials used. Proofing and baking times are shown in Table 3.

Further, the obtained samples of the bun were examined in the accredited laboratory of the Scientific Research Institute of Food Safety at the Almaty Technological University.

### 2.2. Statistical Analysis

The data were analyzed using MS Excel for Windows version 10 Pro, 2010. The data collected during the study were subjected to independent tasting, and questionnaires were conducted to assess the organoleptic characteristics of control and test samples. In the process of analysis, absolute and relative statistical indicators and tabular and graphical methods for presenting the results were used.

## 3. Results and Discussion

At the beginning of the work, the physicochemical and microbiological indicators of the raw materials used were investigated. The results of the study of the physicochemical and microbiological indicators of dry sugar beet powder are shown in Table 4.

Table 4 shows that the physicochemical indicators of dried sugar beet were good; in particular, it contained about 69.74% reducing sugars. Moreover, the safety indicators did not exceed the established norms according to regulatory documents (TR CU 021/2011…, 2019).

The results of the study of the physicochemical and microbiological indicators of the first-grade flour are shown in Table 5.

The physical and chemical indicators of the first-grade flour presented in Table 5 correspond to the standards and requirements of GOST 26574-2017 (GOST 26574-2017..., 2019). Moreover, the safety indicators do not exceed the established norms according to regulatory documents (TR CU 021/2011…, 2019).

To determine the optimal formulations, the organoleptic properties of buns of various compositions were determined. The results are shown in Table 6 and Figures 2–12.

**Figure 2:**
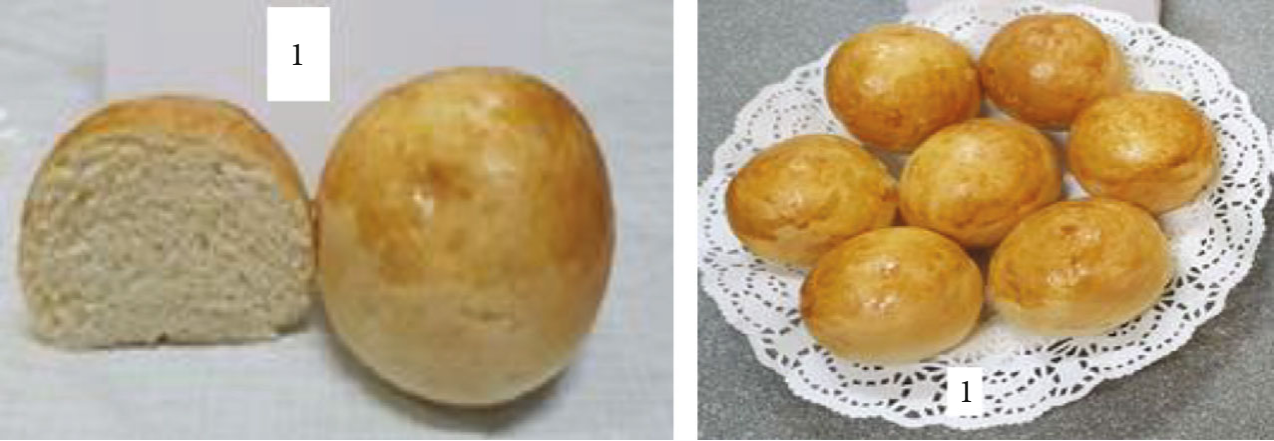
Sample no. 1: control sample with sugar.

**Figure 3:**
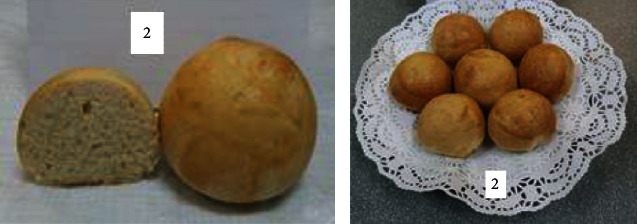
Sample no. 2 with 30 g of sugar beet powder added.

**Figure 4:**
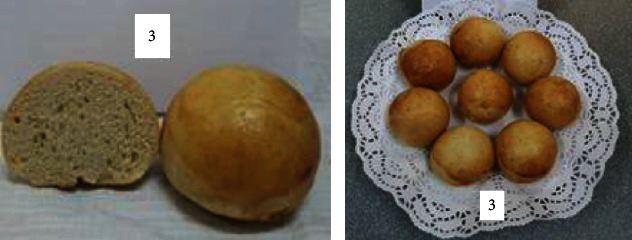
Sample no. 3 with 40 g of sugar beet powder added.

**Figure 5:**
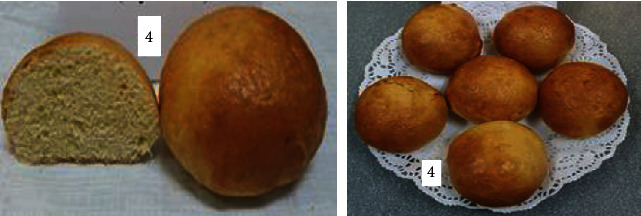
Sample no. 4 made from 15% chickpea flour with sugar.

**Figure 6:**
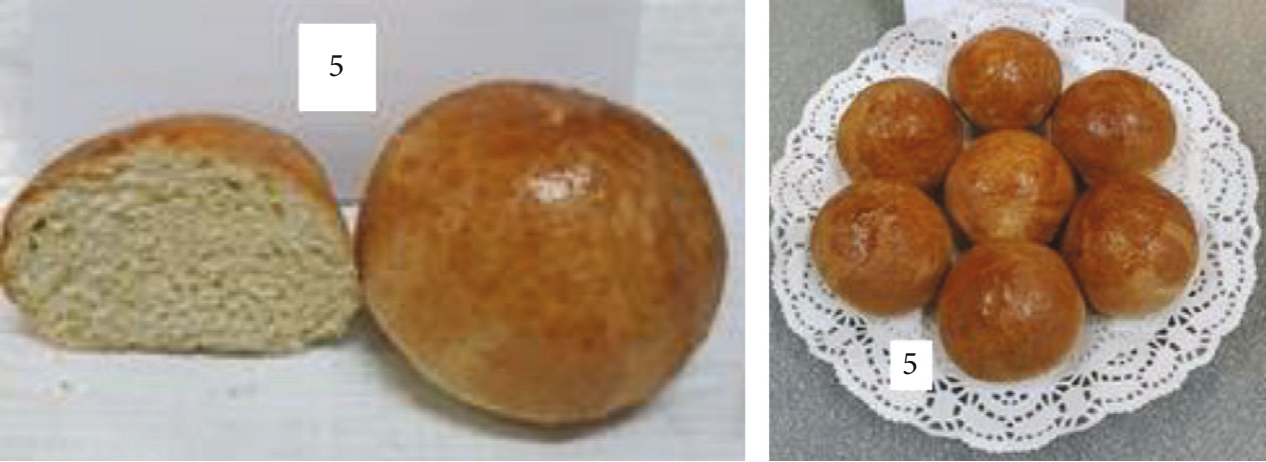
Sample no. 5 made from 15% flour with sugar.

**Figure 7:**
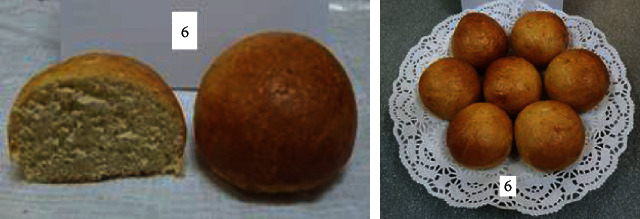
Sample no. 6 made from 15% chickpea flour and 40 g of sugar beet powder.

**Figure 8:**
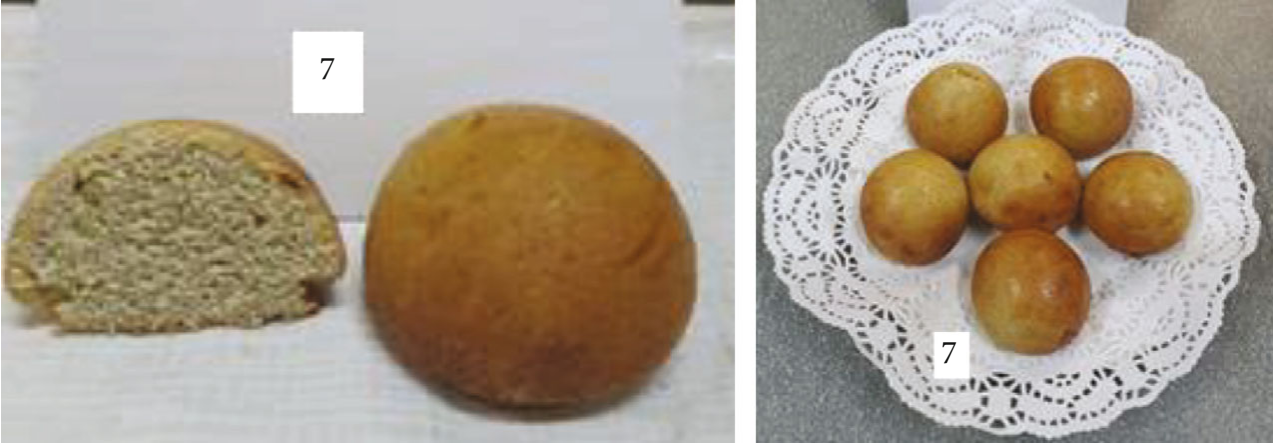
Sample no. 7 made from 15% mung flour and 50 g of sugar beet powder.

**Figure 9:**
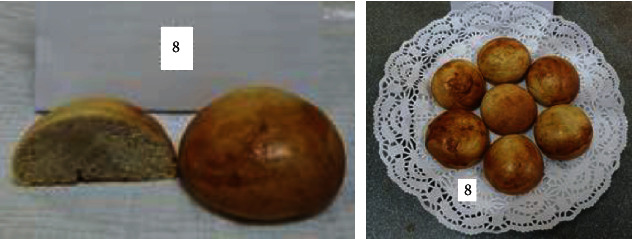
Sample no. 8 made from 15% chickpea and 10% mung flour with sugar.

**Figure 10:**
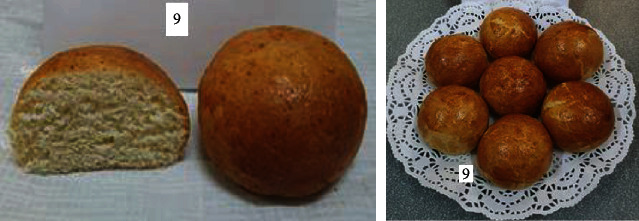
Sample no. 9 made from 5% muffin and 10% chickpea flour with 30 g of sugar beet powder added.

**Figure 11:**
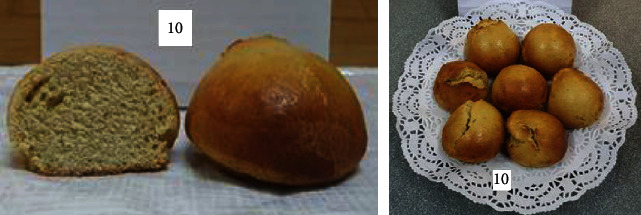
Sample no. 10 made from 5% muffin and 15% chickpea flour with 40 g of sugar beet powder added.

**Figure 12:**
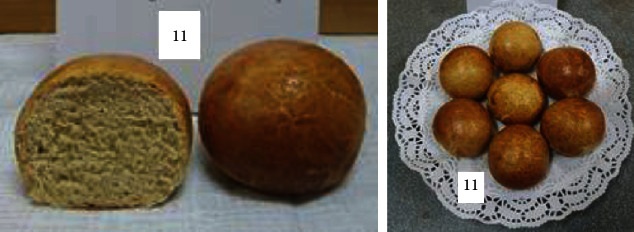
Sample no. 11 made from 5% mung flour and 5% chickpea flour with 40 g of sugar beet powder added.

From the data in Table 6 and Figures 2–12, the following conclusion can be drawn: sample nos. 4, 5, 6, and 9 did not deviate from the control sample (no. 1) and showed better results in terms of organoleptic indicators. In terms of organoleptic indicators, there were also the following disadvantages: sample nos. 2 and 3 were not elastic. Sample no. 7 had an uneven light brown colour, and the crumb was not baked, not elastic, compacted, and firm. Sample no. 8 was uneven in colour, the crumb not baked, damp to the touch, and not elastic. Sample no. 10 had a surface with an undermining, uneven shape, undeveloped porosity, and emptiness, sample no. 11 had an uneven shape, and the porosity was not developed. Chickpeas added volume to the bun, and mung helped to reduce the time of proofing and dough preparation. Based on these results, the following samples of buns were selected for the study of physical and chemical indicators and safety indicators: nos. 1–6 and no. 9.

**Figure 13:**
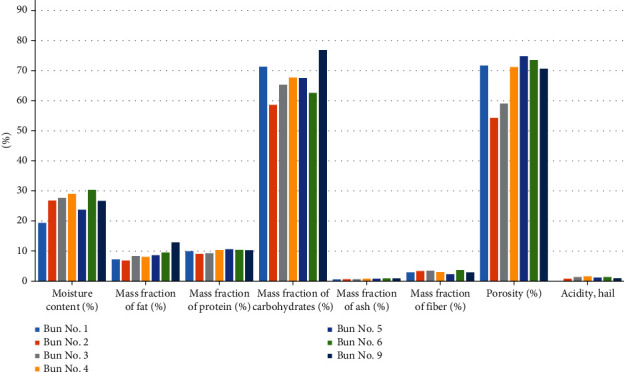
Physical and chemical indicators of various bun formulations.

**Figure 14:**
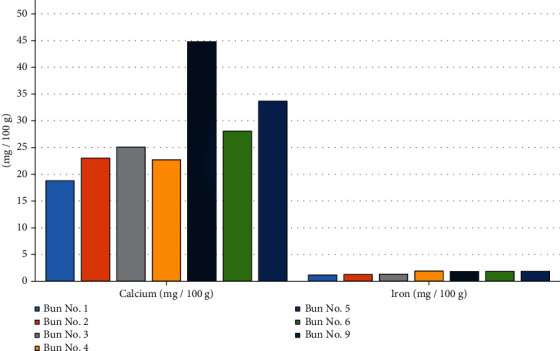
The content of calcium and iron in the composition of the various bun formulations.

**Figure 15:**
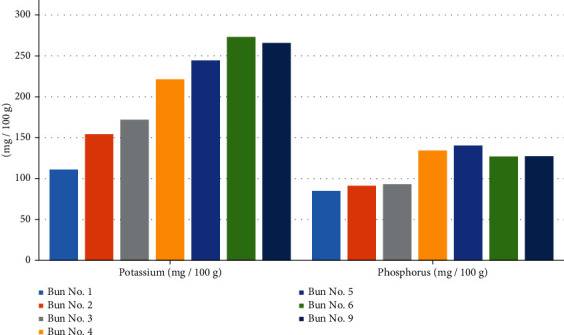
The content of potassium and phosphorus in the various bun formulations.

**Figure 16:**
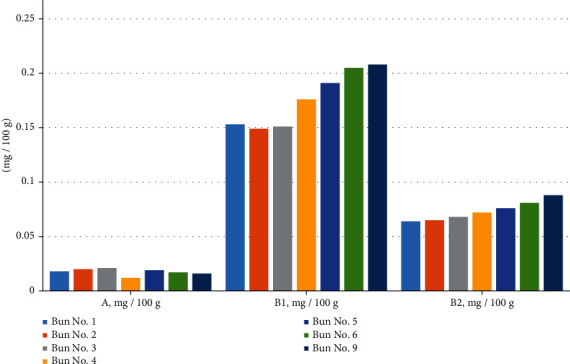
Content of vitamins A, B1, and B2 in the various bun formulations.

**Figure 17:**
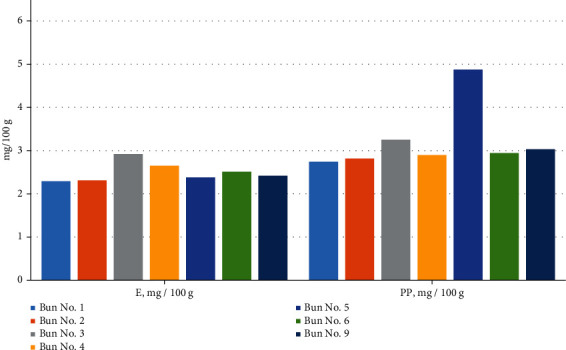
The content of vitamins E and PP in the various bun formulations.

Further, the physicochemical and safety indicators were investigated. The results are shown in Tables 7–10 and Figures 13–17.

To exclude sugar from the bun recipe, dry sugar beet powder was added, and chickpea and muffin flour were also incorporated to enrich the dough with minerals, vitamins, and other nutrients. For comparison, buns prepared with dry sugar beet powder, chickpea, and muffin flour added separately in different ratios and quantities were studied to determine their amounts in the final product.

From the data in Table 7 and Figure 13, it can be seen that, in comparison with the control sample, the moisture content of sample nos. 4 and 6 is 3%–4% higher. This indicates that chickpea flour absorbs more moisture, so it is necessary to reduce the amount of wheat flour in the recipe. Sample no. 5 showed the lowest moisture content as whipped flour helps to improve the consistency of the dough. In terms of the fat mass fraction, buns made of composite flour had the highest value. The protein content of sample nos. 4–6 and no. 9 was 1.0%–1.5% higher. The mass fraction of protein in the control sample was 9.94%, and that in sample no. 9 was equal to 10.29%. Chickpea and mung bean are rich in carbohydrates and fibre, respectively, and buns made with chickpea and mung flour are thus rich in carbohydrates and fibre. The mass fraction of ash in the control sample was 0.58% and in sample no. 9 0.90%. The ash content in dry sugar beet powder, chickpea, and mung flour is very high; therefore, according to Table 7 and Figure 13, all buns, except for the control sample, have a high ash content. The acidity of the control sample is equal to 1.2 degrees, and that in sample no. 9 is equal to 1.0 degrees.

From the data in Table 8 and Figure 14, it can be seen that sample no. 5, made from 15% flour with sugar, has a high calcium content in comparison with the other samples. This indicates that mashed flour helps to increase the calcium content in the bun. The calcium content in sample no. 1 (control sample) was 18.79 mg/100 g, and that in sample no. 9, made from 5% muffin and 10% chickpea flour with 30 g of sugar beet powder added, is 33.67 mg/100 g. The iron content of all tested bun samples is higher than that in the control bun sample, which indicates that dried sugar beet powder, chickpea flour, and muffin flour contain sufficient amounts of iron and contribute to an increase in the iron content of a bun. The iron content of sample no. 1 (control sample) is 1.17 mg/100 g, and that of sample no. 9 is equal to 1.85 mg/100 g.

From the data in Table 8 and Figure 15, it can be seen that the content of potassium and phosphorus in all bun samples is higher than that in the control bun sample. The potassium content in sample no. 1 was 111.06 mg/100 g, and that in sample no. 9 was more than double, or equal to 265.76 mg/100 g. The phosphorus content in sample no. 1 was 84.82 mg/100 g, and that in sample no. 9 was equal to 127.34 mg/100 g.

In Table 9 and Figure 16, it can be seen that the vitamin A content did not differ by much; that is, in sample no. 1, it was 0.018 mg/100 g, and in sample no. 9, it was slightly less, equal to 0.016 mg/100 g. Chickpea and muffin flour are rich in vitamins B1 and B2; therefore, when they were added to the bun recipe, the content of these vitamins increased accordingly. The amount of vitamin B1 was 0.153 mg/100 g in sample no. 1 and 0.208 mg/100 g in sample no. 9, and the vitamin B2 content was 0.064 mg/100 g in sample no. 1 and 0.088 mg/100 g in sample no. 9.

Vitamin E is found in large quantities in sugar beet powder and chickpea flour; therefore, its amount in sample nos. 3, 4, and 6, respectively, was higher. The content of vitamin E in sample no. 1 was 2.29 mg/100 g, and in sample no. 9, it was equal to 2.42 mg/100 g. Mung flour contains a large amount of vitamin PP; therefore, sample no. 5, made from 15% mung flour with sugar had the highest value of all the samples, about 4.874 mg/100 g. Accordingly, in test sample no. 9, the content of vitamin PP is 3.032 mg/100 g, and in the control sample, it is about 2.743 mg/100 g.

From the data in Table 10, it can be seen that the safety indicators did not exceed the established norms according to regulatory documents (TR CU 021/2011, 2019). In sample nos. 2, 3, and 6, toxic elements were found, but they were within the permissible concentrations. All bun samples were free of mycotoxins and pesticides. According to microbiological indicators, it can be seen that the numbers of mesophilic aerobic and facultatively anaerobic microorganisms (NMAFAnM or total microbial number, TMN) in sample nos. 5, 6, and 9 were less than those in the control bun sample. *Escherichia coli* bacteria (colibacillus) were not detected in any bun samples tested. Yeasts were found in sample no. 4, made from 15% chickpea flour with sugar, equal to 1 CFU/g, but did not exceed the established norms. The amount of mold in control sample no. 1 was 7 CFU/g, while other samples showed less and sample nos. 4 and 9 showed none.

**Figure 18:**
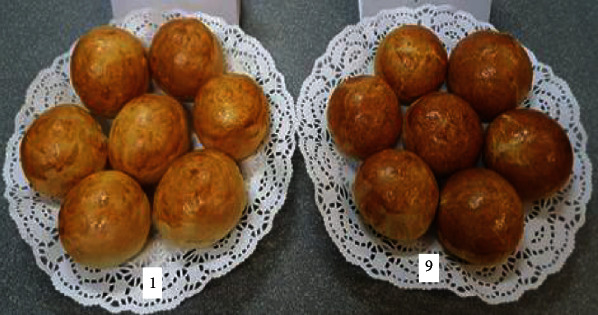
Sample no. 1: control sample and sample no. 9: the best option.

By comparing the various bun formulations, we were able to choose the optimal one. The optimal and most effective option for baking buns is the recipe for sample no. 9, made from 5% mung flour and 10% chickpea flour with the addition of 30 g sugar beet powder (for one portion of laboratory baked goods). In this recipe, dry sugar beet powder completely replaces sugar, and this bun is enriched with useful properties, vitamins, and minerals. Therefore, it is proposed for production. A comparative photo is shown in Figure 18.

Figure 18 shows that the outer colour and shape of the product are improved in sample no. 9 in comparison with the control sample.

The use of mung flour and chickpea flour for making buns shortens the baking and fermentation time and promotes the fermentation process. Dry sugar beet powder reduces the spreading of the dough pieces.

## 4. Conclusion

Recipes and technological regimes for the reception of bakery and flour confectionery products using sugar beet powder and leguminous crops have been developed. Assortments of bakery and pastry products were obtained using sugar beet powder and composite flour of leguminous crops. In order to exclude sugar from the recipe, sugar beet powder is used in the production of buns. In dry sugar powder, the concentration is about 70% sugar and 7.1% moisture. Compared with the recipe for sugar buns, in the recipe for bakery and flour confectionery products with sugar beet powder, a difference in the amount of premium and first-grade flour is established. Pulse flours, especially chickpea flour, have also been found to be highly absorbent. For this sample, first-grade flour was added depending on the consistency of the dough.

As a result, it was determined that sample no. 9 is made according to the following recipe (per 100 g): first-grade wheat flour, -73.23 g; chickpea flour, 10 g; puree flour, 5 g; milk, 46.15 ml; dry yeast, 1.85 g; eggs, 15.39 g; dry sugar beet powder, 9.23 g; and butter, 15.39 g with waiting and proofing time about 95 minutes and waiting time 10 minutes.

## Figures and Tables

**Table 1:** Recipes of the control sample and new assortments of buns, calculated for one portion of laboratory baked goods.

No.	Raw materials	Raw material quantity										
		Sample no. 1	Sample no. 2	Sample no. 3	Sample no. 4	Sample no. 5	Sample no. 6	Sample no. 7	Sample no. 8	Sample no. 9	Sample no. 10	Sample no. 11
1	Flour of the first grade (g)	325	325	325	276.25	276.25	230	178	245	238	225	290
2	Chickpea flour (g)	0	0	0	48.75	0	48.75	48.75	48.75	32.5	48.75	16.25
3	Masha flour (g)	0	0	0	0	48.75	0	0	32.5	16.25	16.25	16.25
4	Milk (ml)	150	150	150	150	150	150	150	150	150	150	150
5	Yeast (g)	6	6	6	6	6	6	6	6	6	6	6
6	Egg (g) (1 piece = 50 g)	50	50	50	50	50	50	50	50	50	50	50
7	Sugar (g)	50	0	0	50	50	0	0	50	0	0	0
8	Dry powder of sugar beet (g)	0	30	40	0	0	40	50	0	30	40	40
9	Butter (g)	50	50	50	50	50	50	50	50	50	50	50
Total		631	611	621	631	631	574.75	532.75	632.25	572.75	586	618.5

**Table 2:** Recipes of the control sample and new assortments of buns, calculated for 100 g of flour.

No.	Raw materials	Raw material quantity										
		Sample no. 1	Sample no. 2	Sample no. 3	Sample no. 4	Sample no. 5	Sample no. 6	Sample no. 7	Sample no. 8	Sample no. 9	Sample no. 10	Sample no. 11
1	Flour of the first grade (g)	100	100	100	85	85	70.77	54.77	75.39	73.23	69.23	89.23
2	Chickpea flour (g)	0	0	0	15	0	15	15	15	10	15	5
3	Masha flour (g)	0	0	0	0	15	0	0	10	5	5	5
4	Milk (ml)	46.15	46.15	46.15	46.15	46.15	46.15	46.15	46.15	46.15	46.15	46.15
5	Yeast (g)	1.85	1.85	1.85	1.85	1.85	1.85	1.85	1.85	1.85	1.85	1.85
6	Egg (g) (1 piece = 50 g)	15.39	15.39	15.39	15.39	15.39	15.39	15.39	15.39	15.39	15.39	15.39
7	Sugar (g)	15.39	0	0	15.39	15.39	0	0	15.39	0	0	0
8	Dry powder of sugar beet (g)	0	9.23	12.31	0	0	12.31	15.38	0	9.23	12.31	12.31
9	Butter (g)	15.39	15.39	15.39	15.39	15.39	15.39	15.39	15.39	15.39	15.39	15.39

**Table 3:** Proofing and baking times of the control sample and new assortments of buns.

Sample	Time, min				
	Dough	Proofing no. 1	Proofing no. 2	Baking	Total
Sample no. 1	15	60	30	10	115
Sample no. 2	15	60	40	10	125
Sample no. 3	15	70	50	13	148
Sample no. 4	15	60	30	10	115
Sample no. 5	15	45	25	9	94
Sample no. 6	15	70	15	12	112
Sample no. 7	15	80	30	10	135
Sample no. 8	15	80	25	12	132
Sample no. 9	15	60	20	10	105
Sample no. 10	15	80	30	12	137
Sample no. 11	15	80	30	11	136

**Table 4:** Physicochemical and microbiological parameters of dry sugar beet powder.

Name of indicators, units of measurement	Actual results
Moisture content (%)	7.09 ± 0.03
Mass fraction of fat (%)	Not found
Mass fraction of protein (%)	1.32 ± 0.1
Mass fraction of carbohydrates (sugar) (%)	69.74 ± 0.1
Mass fraction of ash (%)	1.59 ± 0.005
Mass fraction of fibre (%)	10.75 ± 0.02
Acidity (hail)	1.6 ± 0.1
*Mineral elements*	
Potassium (mg/100 g)	884.15 ± 7.07
Calcium (mg/100 g)	159.03 ± 1.91
Iron (mg/100 g)	6.02 ± 0.02
Phosphorus (mg/100 g)	184.81 ± 1.29
*Vitamins*	
А (mg/100 g)	0.008 ± 0.0002
Е (mg/100 g)	0.41 ± 0.001
В1 (mg/100 g)	0.011 ± 0.002
В2 (mg/100 g)	0.020 ± 0.008
РР (mg/100 g)	1.722 ± 0.344
*Toxic elements*	
Cadmium (mg/kg)	0.0075 ± 0.009
Lead (mg/kg)	0.0137 ± 0.002
*Mycotoxins*	
Aflatoxin B1 (mg/kg)	Not found
*Pesticides*	
Heptachlor (mg/kg(	Not found
Hexachlorocyclohexane (HCCH—*α*-, *β*-, and *γ*-isomers) (mg/kg)	Not found
DDT and its metabolites, mg/kg	Not found
*Microbiological indicators*	
NMAFAnM (CFU/g)	6∗10^3^
BС (coliforms) in 1.0 g of product (CFU/g)	Not found
Yeast (CFU/g)	2
Mold (CFU/g)	2

Note: ±: standard deviation.

**Table 5:** Physicochemical and microbiological indicators of the first-grade flour.

Name of indicator, units of measurement	Actual results
Moisture content (%)	7.37 ± 0.07
Mass fraction of fat (%)	1.28 ± 0.05
Mass fraction of protein (%)	12.96 ± 0.02
Mass fraction of carbohydrates (sugar) (%)	68.64 ± 1.00
Mass fraction of ash (%)	0.59 ± 0.005
Mass fraction of fibre (%)	3.26 ± 0.02
Acidity (hail)	1.0 ± 0.1
*Mineral elements*	
Potassium (mg/100 g)	197.39 ± 2.17
Calcium (mg/100 g)	31.44 ± 0.33
Iron (mg/100 g)	2.09 ± 0.02
Phosphorus (mg/100 g)	135.66 ± 1.49
*Vitamins*	
А (mg/100 g)	0.008 ± 0.0002
Е (mg/100 g)	0.41 ± 0.001
В1 (mg/100 g)	0.011 ± 0.002
В2 (mg/100 g)	0.020 ± 0.008
РР (mg/100 g)	1.722 ± 0.344
*Toxic elements*	
Cadmium (mg/kg)	0.0008 ± 0.0001
Lead (mg/kg)	0.0008 ± 0.0001
*Mycotoxins*	
Aflatoxin B1 (mg/kg)	Not found
*Pesticides*	
Heptachlor (mg/kg)	Not found
Hexachlorocyclohexane (HCCH—*α-*, *β-*, and *γ*-isomers) (mg/kg)	Not found
Dichlorodiphenyl trichloromethylmethane (DDT) and its metabolites (mg/kg)	Not found
*Microbiological indicators*	
NMAFAnM (CFU/g)	7∗10^4^
BС (coliforms) in 1.0 g of product (CFU/g)	Not found
Yeast (CFU/g)	1
Mold (CFU/g)	17

Note: ±: standard deviation.

**Table 6:** Organoleptic characteristics of the control sample and new bun formulations.

Sample	Colour	Smell	Surface	Taste	Form	Crumb condition		
						Baked	Promes	Porosity
Sample no. 1	Light yellow	Specific to this type of product	Glossy, nondestructive	Specific to this type of product	Rounded, not vague, and without imprints	Baked, not wet to touch, and elastic	No lumps and traces of impurities	Developed, void-free, and compacted
Sample no. 2	Light brown	Characteristic of this type of product with a pronounced smell of sugar beet	Glossy, nondestructive	Characteristic of this type of product with a pronounced taste of sugar beet	Rounded, not vague, and without imprints	Baked, not wet to the touch, and not elastic	No lumps and traces of impurities	Developed, void-free, and compacted
Sample no. 3	Light brown	Characteristic of this type of product with a pronounced smell of sugar beet	Glossy, nondestructive	Characteristic of this type of product with a pronounced taste of sugar beet	Rounded, not vague, and without imprints	Baked, not wet to the touch, and not elastic	No lumps and traces of impurities	Developed, void-free, and compacted
Sample no. 4	Light yellow	Specific to this type of product	Glossy, nondestructive	Specific to this type of product	Rounded, not vague, and without imprints	Baked, not wet to touch, and elastic	No lumps and traces of impurities	Developed, void-free, and compacted
Sample no. 5	Brown	Specific to this type of product	Glossy, nondestructive	Specific to this type of product	Rounded, slightly vague, and without imprints	Baked, not wet to touch, and elastic	No lumps and traces of impurities	Developed, void-free, and compacted
Sample no. 6	Light brown	Specific to this type of product	Glossy, nondestructive	Specific to this type of product	Rounded, not vague, and without imprints	Baked, not wet to touch, and elastic	No lumps and traces of impurities	Developed, void-free, and compacted
Sample no. 7	Not even, light brown	Specific to this type of product	Glossy, with undermining	Specific to this type of product	Not even	Not baked, not wet to the touch, and not elastic	No lumps and traces of impurities	Compacted, solid
Sample no. 8	Yellow, not even	Specific to this type of product	Glossy, nondestructive	Specific to this type of product	Rounded, not vague, and without imprints	Not baked, damp to the touch, and not elastic	No lumps and traces of impurities	Developed, void-free, and compacted
Sample no. 9	Brown	Specific to this type of product	Glossy, nondestructive	Specific to this type of product	Rounded, not vague, and without imprints	Baked, not wet to touch, and elastic	No lumps and traces of impurities	Developed, void-free, and compacted
Sample no. 10	Light brown	Specific to this type of product	Glossy, with undermining	Specific to this type of product	Rounded, not even, and without imprints	Baked, not wet to touch, and elastic	No lumps and traces of impurities	Undeveloped, with emptiness, and compacted
Sample no. 11	Light brown	Specific to this type of product	Glossy, nondestructive	Specific to this type of product	Rounded, not even, and without imprints	Baked, not wet to touch, and elastic	No lumps and traces of impurities	Undeveloped, void-free, and compacted

Note: Promes: the state of the crumb of a bakery product, characterized by the absence of unmixed raw materials.

**Table 7:** Physical and chemical indicators of various bun formulations.

Name of indicators, units of measurement	Actual results						
	Sample no. 1	Sample no. 2	Sample no. 3	Sample no. 4	Sample no. 5	Sample no. 6	Sample no. 9
Moisture content (%)	25.38 ± 0.03	26.77 ± 0.04	27.68 ± 0.1	28.98 ± 0.05	23.76 ± 0.12	30.36 ± 0.1	26.72 ± 0.1
Mass fraction of fat (%)	7.25 ± 0.04	6.84 ± 0.05	8.34 ± 0.03	8.09 ± 0.2	8.64 ± 0.02	9.49 ± 0.03	12.91 ± 0.1
Mass fraction of protein (%)	9.94 ± 0.1	9.06 ± 0.05	9.30 ± 0.1	10.35 ± 0.1	10.65 ± 0.1	10.41 ± 0.04	10.29 ± 0.04
Mass fraction of carbohydrates (%)	71.30 ± 1.07	58.60 ± 0.50	65.29 ± 1.01	67.66 ± 0.70	67.51 ± 1.02	62.55 ± 0.80	76.81 ± 0.48
Mass fraction of ash (%)	0.58 ± 0.005	0.66 ± 0.006	0.68 ± 0.007	0.82 ± 0.009	0.81 ± 0.007	0.95 ± 0.009	0.90 ± 0.008
Mass fraction of fibre (%)	2.91 ± 0.03	3.37 ± 0.03	3.52 ± 0.02	3.01 ± 0.03	2.32 ± 0.01	3.71 ± 0.02	2.93 ± 0.01
Porosity (%)	71.67 ± 0.88	54.26 ± 0.81	59.06 ± 0.89	71.17 ± 0.78	74.72 ± 1.12	73.47 ± 0.81	70.57 ± 0.78
Acidity (hail)	1.2	0.8	1.4	1.6	1.2	1.4	1.0

Note: ±: standard deviation.

**Table 8:** Mineral elements of various bun formulations.

Name of indicators, units of measurement	Actual results						
	Sample no. 1	Sample no. 2	Sample no. 3	Sample no. 4	Sample no. 5	Sample no. 6	Sample no. 9
Calcium (mg/100 g)	18.79 ± 0.28	23.02 ± 0.18	25.09 ± 0.28	22.72 ± 0.30	44.77 ± 0.49	28.08 ± 0.22	33.67 ± 0.44
Iron (mg/100 g)	1.17 ± 0.01	1.25 ± 0.01	1.34 ± 0.01	1.93 ± 0.02	1.9 ± 0.02	1.87 ± 0.02	1.85 ± 0.02
Potassium, mg/100 g	111.06 ± 1.22	154.31 ± 1.7	171.99 ± 1.55	221.30 ± 2.88	244.41 ± 4.15	273.09 ± 4.09	265.76 ± 3.99
Phosphorus (mg/100 g)	84.82 ± 0.76	91.34 ± 0.70	93.12 ± 0.74	134.24 ± 1.75	140.24 ± 1.26	126.85 ± 1.40	127.34 ± 1.40

Note: ±: standard deviation.

**Table 9:** Vitamin composition of various bun formulations.

Name of indicators, units of measurement	Actual results						
	Sample no. 1	Sample no. 2	Sample no. 3	Sample no. 4	Sample no. 5	Sample no. 6	Sample no. 9
- А (mg/100 g)	0.018 ± 0.0002	0.020 ± 0.0001	0.021 ± 0.0002	0.012 ± 0.0001	0.019 ± 0.0001	0.017 ± 0.0002	0.016 ± 0.0002
- В1 (mg/100 g)	0.153 ± 0.031	0.149 ± 0.030	0.151 ± 0.030	0.176 ± 0.028	0.191 ± 0.031	0.205 ± 0.038	0.208 ± 0.042
- В2 (mg/100 g)	0.064 ± 0.027	0.065 ± 0.027	0.068 ± 0.029	0.072 ± 0.011	0.076 ± 0.029	0.081 ± 0.024	0.088 ± 0.037
- Е (mg/100 g)	2.29 ± 0.005	2.31 ± 0.004	2.92 ± 0.002	2.65 ± 0.002	2.38 ± 0.001	2.51 ± 0.002	2.42 ± 0.001
- РР (mg/100 g)	2.743 ± 0.089	2.817 ± 0.063	3.253 ± 0.051	2.898 ± 0.017	4.874 ± 0.074	2.942 ± 0.057	3.032 ± 0.006

Note: ±: standard deviation.

**Table 10:** Safety indicators of assortments of buns.

Name of indicators, units of measurement	Actual results						
	Sample no. 1	Sample no. 2	Sample no. 3	Sample no. 4	Sample no. 5	Sample no. 6	Sample no. 9
*Toxic elements*							
Cadmium (mg/kg)	Not found	0.0008 ± 0.00001	0.0011 ± 0.00001	Not found	Not found	Not found	Not found
Lead (mg/kg)	Not found	0.0012 ± 0.00002	0.0017 ± 0.00001	Not found	Not found	0.0010 ± 0.00001	Not found
*Mycotoxins*							
Aflatoxin B1 (mg/kg)	Not found	Not found	Not found	Not found	Not found	Not found	Not found
*Pesticides*							
Heptachlor (mg/kg)	Not found	Not found	Not found	Not found	Not found	Not found	Not found
Hexachlorocyclohexane (HCCH—*α-*, *β-*, and *γ*-isomers) (mg/kg)	Not found	Not found	Not found	Not found	Not found	Not found	Not found
Dichlorodiphenyl trichloromethylmethane (DDT) and its metabolites (mg/kg)	Not found	Not found	Not found	Not found	Not found	Not found	Not found
*Microbiological indicators*							
NMAFAnM (CFU/g)	7∗10^3^	12∗10^3^	9∗10^3^	11∗10^3^	5∗10^3^	4∗10^3^	5∗10^3^
BС (coliforms) in 1.0 g of product (CFU/g)	Not found	Not found	Not found	Not found	Not found	Not found	Not found
Yeast (CFU/g)	Not found	Not found	Not found	1	Not found	Not found	Not found
Mold (CFU/g)	7	4	3	Not found	2	4	Not found

Note: ±: standard deviation.

## Data Availability

The [DATA TYPE] data used to support the findings of this study are included within the article.

## Additional Points

*Recommendations*. Based on the results obtained, we can draw a conclusion about the relevance of this topic in the field of obtaining a new assortment of bakery products with increased nutritional value. The developed highly efficient technology for producing buns using composite flour and dry sugar beet powder makes it possible to activate biotechnological production processes and save wheat flour; it is also recommended for use in the production of bakery products.
